# Image Captioning Based on Semantic Scenes

**DOI:** 10.3390/e26100876

**Published:** 2024-10-18

**Authors:** Fengzhi Zhao, Zhezhou Yu, Tao Wang, Yi Lv

**Affiliations:** 1 College of Computer Science and Technology, Jilin University, Changchun 130012, China; yuzz@jlu.edu.cn (Z.Y.); taowang19@mails.jlu.edu.cn (T.W.); lvyi18@mails.jlu.edu.cn (Y.L.); 2 Key Laboratory of Symbolic Computation and Knowledge Engineering of Ministry of Education, Jilin University, Changchun 130012, China; 3 Guang Dong Peizheng College, Guangzhou 510830, China

**Keywords:** image captioning, semantic scenes encoder, attention mechanism, graph

## Abstract

With the development of artificial intelligence and deep learning technologies, image captioning has become an important research direction at the intersection of computer vision and natural language processing. The purpose of image captioning is to generate corresponding natural language descriptions by understanding the content of images. This technology has broad application prospects in fields such as image retrieval, autonomous driving, and visual question answering. Currently, many researchers have proposed region-based image captioning methods. These methods generate captions by extracting features from different regions of an image. However, they often rely on local features of the image and overlook the understanding of the overall scene, leading to captions that lack coherence and accuracy when dealing with complex scenes. Additionally, image captioning methods are unable to extract complete semantic information from visual data, which may lead to captions with biases and deficiencies. Due to these reasons, existing methods struggle to generate comprehensive and accurate captions. To fill this gap, we propose the Semantic Scenes Encoder (SSE) for image captioning. It first extracts a scene graph from the image and integrates it into the encoding of the image information. Then, it extracts a semantic graph from the captions and preserves semantic information through a learnable attention mechanism, which we refer to as the dictionary. During the generation of captions, it combines the encoded information of the image and the learned semantic information to generate complete and accurate captions. To verify the effectiveness of the SSE, we tested the model on the MSCOCO dataset. The experimental results show that the SSE improves the overall quality of the captions. The improvement in scores across multiple evaluation metrics further demonstrates that the SSE possesses significant advantages when processing identical images.

## 1. Introduction

Image captioning is a task in the field of computer vision which involves the generation of descriptive natural language for images by comprehending the visual information contained within them [1]. Many methodologies for image captioning rely on object detection networks, such as Faster R-CNN [2] and DETR [3], to extract region features from images. The next step in the image captioning process entails the generation of natural language descriptions based on these region features, typically using models such as LSTM [4] and Transformer [5]. Object detection models play a crucial role in identifying and classifying objects within images, providing the foundational region features necessary for generating captions. Meanwhile, natural language generation models have increasingly incorporated attention mechanisms [6], enhancing their ability to focus on relevant features when producing captions.

One of the primary challenges in image captioning lies in the complexity of fully understanding the scenes embedded within an image. However, current mainstream approaches [7] tend to focus on processing region features that are primarily used for object classification [8,9]. While this approach has yielded some success, it has significant limitations in capturing the full spectrum of relationships between objects in a scene [10], often leading to inaccuracies and a lack of depth in the generated captions. Another major challenge in image captioning is the autoregressive decoding methods [11] which predict the current word based on the previous word and input features. Due to the fact that language has both syntactic structure and semantic complexity [12], relying solely on the already generated captions often fails to capture the entire semantic context of the image [13,14]. This approach can result in a superficial understanding of the image, as it depends on sequential word prediction without fully integrating the broader and more complex relationships among objects within the semantics. As a consequence, the generated captions may lack coherence and fail to accurately reflect the nuanced interactions and spatial arrangements present in the image. Recent advancements in image captioning have incorporated scene graphs to better capture the complex relationships within images.

To overcome the existing limitations, we propose the Semantic Scenes Encoder (SSE) method, which integrates both scene graphs and semantic graphs into the image captioning process. Scene graphs are used to represent the spatial relationships between objects within an image, providing a structured and comprehensive understanding of how different elements interact [15,16]. This spatial awareness improves the contextual accuracy of the generated captions by enabling the model to better capture object relationships and interactions [14]. In this work, we leverage Motifs, a method that identifies common patterns in object interactions within images, to generate scene graphs. By capturing these typical interaction patterns, Motifs enhance the accuracy of scene representation. Additionally, SPICE is employed to generate semantic graphs, which convert captions into structured representations of their semantic content. Beyond image-based scene graphs, capturing the semantic relationships within captions is equally important [17]. Semantic graphs provide a systematic representation of the words in a caption, reflecting not only the individual words but also the complex interplay of semantics that arises from their combination. The integration of high-level image features with detailed semantic relationships allows for the generation of more accurate and contextually rich captions [18]. By incorporating both scene and semantic graphs, which capture intricate relationships and higher-level information [19], the quality of generated captions is significantly enhanced [20]. This comprehensive approach to both spatial and semantic information represents a major advancement in the field of image captioning, paving the way for future research and applications. One of the key contributions of this method is its ability to integrate scene graphs and semantic graphs, which effectively enhances the accuracy and contextual relevance of image caption generation. Compared to traditional region-based feature extraction methods, this approach not only generates a scene graph from the image to capture spatial relationships between objects but also uses SPICE to construct a semantic graph, extracting complex semantic information from the captions. By combining both, the model is able to consider both visual information from the image and semantic information from the text when generating captions, resulting in more accurate and richer descriptions. Additionally, the dictionary module, utilizing a self-attention mechanism, stores high-level semantic information, further improving the model’s ability to understand textual context.

**Figure 1:**
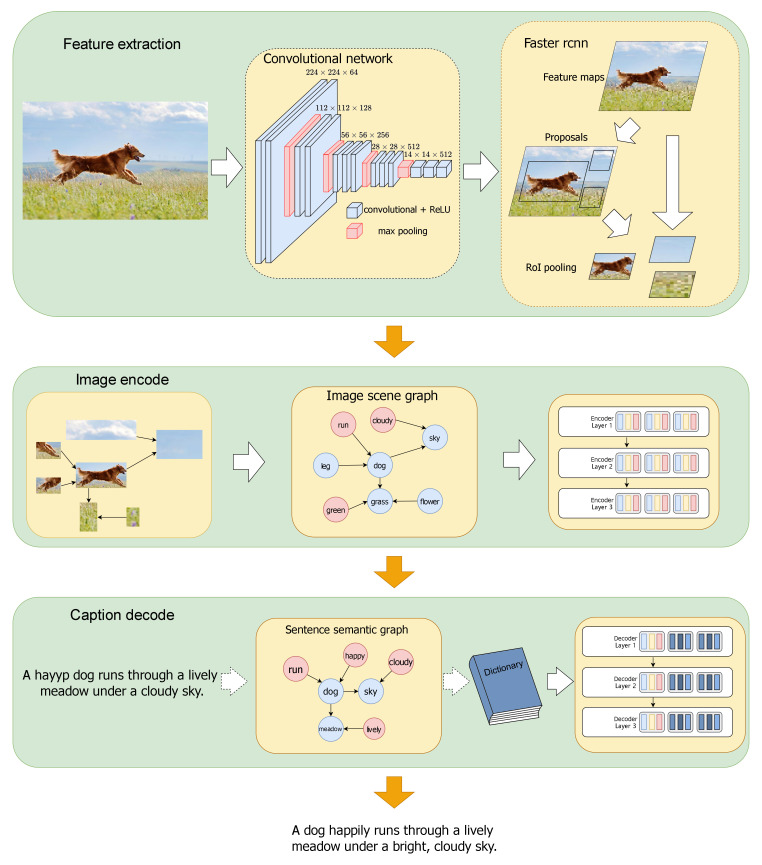
The image illustrates the overall architecture of our proposed method.

Figure 1 illustrates the overall structure of the SSE. The model proposed in this paper is based on DLCT, integrating scene graphs and semantic graphs. The model first preprocesses the input image, using Faster R-CNN to extract region features. Then, during the encoding phase, the SSE uses Motifs [21] to predict relationships between the extracted regions and generate a scene graph. The scene graph and region features are simultaneously input into the image encoder to obtain intermediate representation vectors of the image features. Finally, in the decoding phase, the SSE employs the SPICE [22] method, which was originally designed to evaluate the performance of image captions, to generate a semantic graph. The semantic graph is then used to train a dictionary that stores semantic information. This dictionary serves as a storage module for the semantic information extracted from the graph. The intermediate image representations and stored dictionary contents are all fed into the attention mechanism to produce the final caption. The details will be described in Section 3 of the paper.In summary, the contributions of this paper are as follows:

- The SSE generated two types of graphs: one is the scene graph representing image information extracted through Motifs, and the other is the semantic graph representing the caption constructed using SPICE.
- We constructed a dictionary that uses an attention mechanism to store the high-level semantic information of the captions.
- Through ablation experiments, we validated that incorporating scene graphs into the encoder process surpasses that of previous methods and that the incorporation of semantic graphs also outperforms that of prior approaches.

The structure of the paper is as follows: Section 2 reviews the related work on image captioning, graphs and dictionary units. Section 3 provides a detailed explanation of the SSE. Section 4 describes the specific details of the experiments. In Section 5, we discuss our experimental results and ablation studies. Finally, Section 6 concludes the paper and suggests directions for future research.

## 2. Related Work

In this section, we begin by reviewing methods that incorporate scene graphs in image captioning. These methods represent the objects, attributes, and relationships within an image in a structured way, allowing models to better understand and describe the visual content. Scene graphs are crucial for capturing the spatial and relational aspects of images, which significantly improve the contextual relevance and coherence of the generated captions. Following this, we provide an overview of models that leverage stored information, such as memory modules or dictionaries, to enhance text generation quality. These models utilize previously learned visual-semantic data or textual representations to refine the captioning process.

**Image captioning.** Scene graphs [23,24] provide a structured representation of the objects, attributes, and relationships within a scene, allowing models to better understand and describe the visual content. A semantic graph provides a comprehensive representation of the overall semantic information within captions, ensuring that the generated captions capture the essential meaning [14]. In recent years, the field of image captioning has made significant strides by incorporating such graph-based and semantic information into the training process. By leveraging these semantic structures, models can generate more nuanced and contextually accurate captions, significantly improving the quality of the generated captions by better capturing the intricate relationships and higher-level information within the visual content.

Various methods have been proposed to translate scenes into high-level abstract symbols, facilitating more effective language generation. For example, Gao et al. [25] introduced an approach that derives semantic representations from scene graphs, emphasizing simplicity by avoiding complex image preprocessing. While this method is efficient, it often overlooks the rich contextual relationships present in complex scenes, leading to less comprehensive captions. In contrast, our proposed Semantic Scenes Encoder (SSE) addresses these limitations by integrating not only scene graphs but also semantic graphs into the image captioning process. By capturing both the spatial relationships in the scene and the higher-level semantic relationships of the captions, the SSE provides a more holistic representation of the visual content, leading to more accurate and contextually rich captions. Recent advancements, such as the Attribute-Specific Graph (ASG) framework [26], have improved caption diversity and accuracy by modeling attributes and relationships within the scene. However, these methods often rely solely on scene-specific information, limiting their ability to fully understand the interactions between objects and their semantic meaning within the context of a caption. Our approach goes beyond this by incorporating semantic graphs from captions into the learning process, allowing the model to better capture complex semantic relationships, leading to captions that are not only accurate but also semantically coherent. Additionally, methods like Scene Graph Decomposition (SGD) [27] break down complex scene graphs into smaller, more manageable subgraphs, improving control over specific scene parts. However, these approaches often miss the broader contextual meaning of the image. The SSE, on the other hand, combines scene and semantic graphs, which enables the model to generate captions that reflect both detailed object interactions and overall semantic coherence, surpassing the limitations of subgraph-focused models. The Scene Graph Convolutional (SGC) model [28] and other similar approaches focus on extracting structural-semantic features but tend to overlook the inherent complexity of the captions’ semantic content. By integrating a learnable dictionary module based on the attention mechanism, our SSE model stores high-level semantic information from captions and combines it with the visual representation, improving the model’s ability to generate captions that truly reflect the intricate relationships between objects and their semantic context. Furthermore, while models such as the Scene Graph Visual Storytelling (SGVST) model [29] are designed for sequential image-to-story generation, they lack the fine-grained control over individual image captions that the SSE provides. Our approach can generate more detailed and accurate captions by utilizing both visual and semantic graph information, ensuring that each image’s specific context is accurately captured.

One of the most critical contributions of our work is the use of the SPICE metric [22], which evaluates the semantic content of captions. Unlike traditional metrics like BLEU or ROUGE, SPICE focuses on the propositional content of captions, aligning more closely with human judgment of quality. Our SSE method not only leverages SPICE for evaluation but also integrates semantic graphs directly into the training process, improving the model’s ability to generate captions that capture both syntactic structure and deeper semantic meaning. This combination allows the SSE to outperform existing models, particularly in terms of capturing nuanced relationships within complex scenes.

In summary, the key advantages of our Semantic Scenes Encoder (SSE) approach include its ability to integrate scene and semantic graphs for a more comprehensive understanding of both visual and textual information, the use of a learnable dictionary to store high-level semantic information, and its superior performance in regard to both syntactic and semantic metrics compared to previous methods.

**Dictionary.** In the field of image captioning, the concept of a “dictionary” often refers to a systematic storage module that organizes and stores semantic information. This “dictionary” acts as a memory unit, associating additional context and semantic details with each word or concept within the captions. Significant advancements have been made in integrating memory mechanisms into image captioning systems to enhance their linguistic output and the overall coherence of generated captions.

Cornia et al. [9] introduced a Transformer model with meshed connectivity and memory layers, improving the coherence of and detail in the captions. Similarly, Xu et al. [30] used key–value memory pairs to enhance paragraph-level captions. Further developments, such as dynamic memory modules [31], have enriched caption generation by retrieving visual-semantic data but often fail to fully integrate the complex relationships between image content and captions. Our Semantic Scenes Encoder (SSE) builds on these approaches by combining memory systems with scene and semantic graphs, allowing for adaptive integration of the spatial and semantic relationships. This enables the SSE to generate captions that capture both fine details and broader contextual meaning, addressing limitations in prior models. While attention-focused models, like Guided Visual Attention (GVA) [32] and others [33], improve focus on relevant parts of an image, they often miss the intricate semantic relationships between objects. In contrast, the SSE uses both attention mechanisms and a learnable dictionary to capture high-level semantic information, allowing the model to generate more contextually rich captions. Sheng et al. [34] also explored integrating attention and memory but lacked the adaptability of the SSE’s semantic graphs. By combining memory modules with graph-based representations, the SSE achieves a higher degree of coherence and surpasses traditional methods [35,36].

These developments underscore the evolving landscape of image captioning, where memory systems play a pivotal role in enhancing the descriptive capabilities of models, leading to more accurate and contextually rich textual interpretations of visual data.

## 3. Method

The model’s task is to receive an image and generate captions that accurately describe its content. The model operates in two stages. In the first stage, the image is processed by first extracting region features using Faster R-CNN. These features are then used to construct a scene graph of the regions with the help of Motifs [21]. The region features and the scene graph are subsequently encoded to produce an intermediate representation of the image. The second stage focuses on caption generation. During this stage, SPICE [22] is employed to convert the captions into a semantic graph, which is stored using a dictionary module, referred to as the “dictionary” in the SSE method. Finally, the model generates captions by leveraging the image’s intermediate representation and the information stored in the dictionary.

### 3.1. Problem Formulation

Assume that the image we have is denoted as *I* and the generated caption is denoted as *T*. Image captioning models usually follow a structure consisting of an encoder and a decoder. The encoder E extracts visual features $V=\{v_{1},\ldots ,v_{k}\}$ from the image, where $v_{i}\in R^{d}$ represents some information about the image. Then, the decoder D generates a complete description $T=\{t_{1},\ldots ,t_{n}\}$ based on the obtained image information *V*, where $t_{i}$ represents a word. The process can be represented by the following formula:

(1) V=E(I),T=D(V).

The model employs traditional training methods which consist of two steps. Initially, the model is trained with a word-level cross-entropy (XE) loss function [37]. Subsequently, to improve the generation of words, we apply reinforcement learning [38] for additional refinement. During the first phase, the model learns to predict the next word using the context provided by previously seen actual words. During this process, the decoder takes the input sequence of tokens, enabling the generation of the entire output token sequence in one pass through parallel temporal procedures:

(2) $$L_{XE}\left(\theta \right)=-\sum_{n=1}^{N}\log\left(p_{\theta }\left(t_{n}^{*}|t_{0:n-1}^{*}\right)\right),$$

$t_{0:n}^{*}$ is the ground truth target sequence and *θ* represents the model’s parameters. $p_{\theta }\left(t_{n}^{*}|t_{0:n-1}^{*}\right)$ represents the likelihood of the model producing the next correct word based on the given parameters and all preceding words. The goal of training the model is to minimize the cross-entropy loss function. When the loss function (2) stops decreasing, we employ reinforcement learning techniques [38]. Unlike previous methods [8], our approach uses the average of the rewards as the baseline. The gradient of the model parameters is then adjusted using the difference between the reward of each sampled sequence and the baseline. Specifically, the gradient for a single sample is updated as follows:

(3) $$\nabla_{\theta }=\sum_{i}\left(r(w_{i})-b\right)\nabla_{\theta }\log p_{\theta }\left(w_{i}\right),$$

$w_{i}$ refers to the *i*-th sentence generated during beam search and r(·) stands for the reward function. The baseline *b* is calculated by taking the mean of the rewards obtained from the sampled sequences. This adjustment ensures that sequences with rewards higher than the baseline positively influence the model’s parameters while those with lower rewards have a negative impact. The overall training process leverages these updates to refine the model, ultimately enhancing the quality and accuracy of the generated sentences.

### 3.2. Region Feature

Since the regional features, which include detailed features of objects in the images, are used for subsequent scene graph generation and information encoding, the extraction of these features is performed first. In this experiment, features are extracted using a CNN based on Faster R-CNN [2] with ResNet-101. The intermediate results of ResNet-101 are used as input feature vectors for the Region Proposal Network (RPN), generating bounding boxes for object proposals. Then, non-maximum suppression is applied to discard overlapping bounding boxes. Next, a Region of Interest (RoI) pooling layer is used to convert the remaining bounding boxes to the same spatial size (e.g., 14 × 14 × 2048). Finally, a CNN layer is used to predict the likelihood of objects within these boxes, and the retained objects are average-pooled across the spatial dimensions, thus converting each feature into a 2048-dimensional vector. We serialize these features into $R=[r_{1},\ldots ,r_{n}]$, where $r_{i}=\left[f_{i},p_{i}\right]$ contains the feature vector $f_{i}$ and an object distribution probability of $p_{i}\in R^{\left|C\right|}$. These features are used both for generating image scene graphs and for compiling object information during the caption encoding phase.

### 3.3. Image Scene Graph

Following [21], the experiment converts the image content into a structured graphical representation of the visual scene. A graph G (comprising regions *R*, object labels *L*, and connections *C*) is broken down into the probabilities of three components:

(4) P(G∣I)=P(R∣I)P(L∣R,I)P(C∣R,L,I).

**Figure 2:**
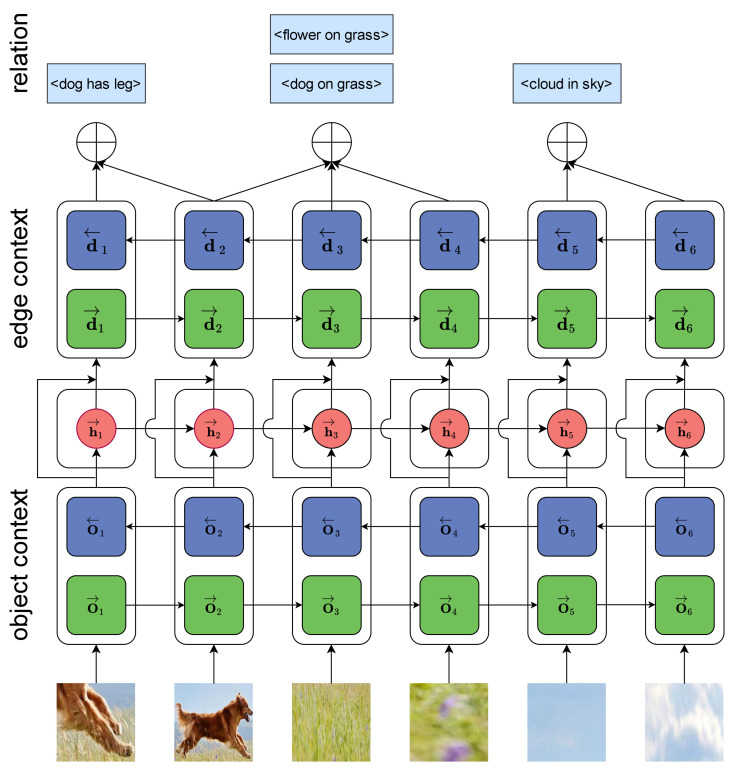
The image depicts the method for constructing an image scene graph.

Importantly, this factorization does not assume any independence. The prediction of object labels can influence one another, and the prediction of relation labels often relies on these object labels. Capturing these dependencies is crucial. In Section 3.2, we obtain the features of the region $R=[r_{1},\ldots ,r_{n}]$ in the image. Each $r_{i}$ comprises a feature vector $f_{i}$ and a vector $p_{i}$, representing the (non-contextualized) probabilities of object labels. Based on Equation (4), we use two-layer Bidirectional Long Short-Term Memory (BiLSTM) to generate the relational attributes of objects. As shown in Figure 2, the first layer is the context layer, which provides the potential distribution information of objects:

(5) $$O=\mathrm{biLSTM}\left({\left[f_{i};W_{1}p_{i}\right]}_{i=1,\ldots ,n}\right),$$

where $O=[o_{1},\ldots ,o_{n}]$ includes the hidden states from the final LSTM layer for each element in the linear sequence of *B*. The parameter matrix $W_{1}$ is used to transform the predicted class distribution. The object context **O** is used to sequentially decode labels for each proposed bounding region by considering the labels decoded in previous steps. An LSTM is utilized to assign a category label to each contextualized representation within **O**.

(6) $$\begin{array}{c} h_{i}={\mathrm{LSTM}}_{i}\left(\left[o_{i};{\hat{o}}_{i-1}\right]\right),\\ {\hat{o}}_{i}=\mathrm{argmax}\left(W_{o}h_{i}\right), \end{array}$$

$\hat{o}$ contains the edge attributes of objects, which are used for the probability distribution of objects in the relational context. The second layer is the edge context layer, which captures the edge information of objects and predicts the positional relationships between objects:

(7) $$D=\mathrm{biLSTM}\left({\left[o_{i};W_{2}{\hat{o}}_{i}\right]}_{i=1,\ldots ,n}\right),$$

$D=[d_{1},\ldots ,d_{n}]$ holds the final layer states for each bounding region, with $W_{2}$ being a parameter matrix. There are *n* objects here, so there are a total of $n^{2}$ possible relationships. The likelihoods of these relationships are calculated using the following formula:

(8) $$\begin{array}{c} g_{i,j}=\left(W_{i}d_{i}\right)\circ \left(W_{j}d_{j}\right)\circ f_{i,j},\\ r_{i,j}=\mathrm{softmax}\left(W_{r}g_{i,j}+w_{o_{i},o_{j}}\right), \end{array}$$

where $d_{i},d_{j}\in D$, $W_{i}$, $W_{j}$ are the learned parameters, $w_{o_{i},o_{j}}$ is the bias vector, and $f_{i,j}$ is a feature vector for the union of boxes. Next, a simple fully connected layer is used to obtain the attributes of the objects.

(9) $$a_{i,l}=\mathrm{softmax}\left(W_{a}d_{i}+b_{a}\right),$$

$W_{a}$ is the learned parameter for generating object attributes and $b_{a}$ is the bias. The final construction of a scene graph containing relationships $r_{i,j}$, attributes $a_{i,l}$, and objects $o_{i}$ is completed.

The constructed image scene graph cannot be used directly. We need to jointly encode it with the regional features to obtain the encoded features. The scene graph of the image mainly includes three categories: objects, the relationships between them, and the attributes of the objects. First, these need to be encoded from one-hot vectors to embedded vectors: $e_{o_{i}}$ (object *i*), $e_{r_{i,j}}$ (relationship between objects *i* and *j*), and $e_{a_{i,l}}$ (attribute *l* of object *i*). When the relational triplet <object, relationship, subject> is obtained, it is fused through a fully connected layer:

(10) $$x_{r_{ij}}=g_{r}\left(e_{o_{i}},e_{r_{i,j}},e_{o_{j}}\right),$$

$g_{r}$ is a fully connected neural network layer and $e_{o_{i}}$ and $e_{o_{j}}$ are the subject and object, respectively. After concatenating the three types of features, the relationship features of the scene graph are generated through this layer. For the attributes of node $o_{i}$, we calculate the feature vectors of its attributes and then compute their average:

(11) $$x_{a_{i}}=\frac{1}{Na_{i}}\sum_{l=1}^{Na_{i}}g_{a}\left(e_{o_{i}},e_{a_{i,l}}\right),$$

$g_{a}$ is a fully connected neural network layer that converts the scene graph features into encoded features. In a scene graph, an entity may act as both a subject and an object. Therefore, distinct functions should be employed to integrate this information. To prevent ambiguity in the interpretation of the same “predicate” across various contexts, it is essential to incorporate the knowledge of the complete relationship triplets involving $o_{i}$ into $x_{o_{i}}$.

(12) $$x_{o_{i}}=\frac{1}{Nr_{i}}\left[\sum_{o_{j}\in sbj\left(o_{i}\right)}g_{s}\left(e_{o_{i}},e_{r_{ij}},e_{o_{j}}\right)+\sum_{o_{k}\in obj\left(o_{i}\right)}g_{o}\left(e_{o_{k}},e_{r_{ki}},e_{o_{i}}\right)\right],$$

where $o_{j}\in sbj\left(o_{i}\right)$ acts as “object” while $o_{i}$ acts as “subject” and $o_{k}\in obj\left(o_{i}\right)$ acts as “subject” while $o_{i}$ acts as “object”. $Nr_{i}=\left|sbj(o_{i})\right|+\left|obj(o_{i})\right|$ represents the count of relationship triplets that include $o_{i}$. At this point, $x_{o_{i}}$, $x_{r_{ij},}$ and $x_{a_{i}}$ integrate the object categories, attributes, and relational features. By concatenating them into $X=[x_{o_{i}},x_{r_{ij}},x_{a_{i}}]$, the final encoding of the image scene graph is achieved.

### 3.4. Semantic Graph

**Figure 3:**
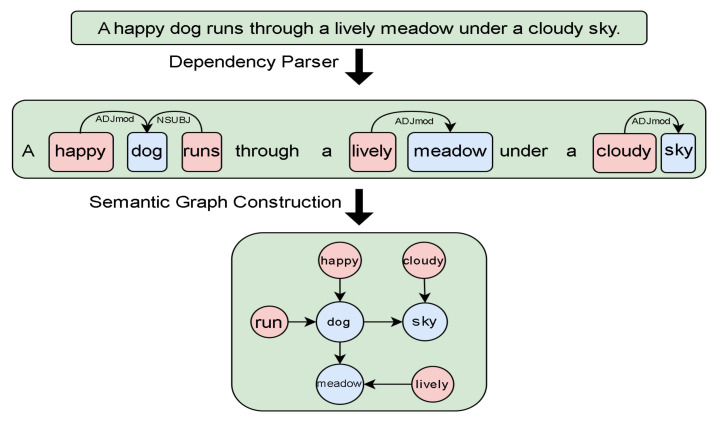
First, use a dependency parser to decompose the sentence into syntactic dependencies [41] between words. Then, use the syntactic dependencies between words to create a semantic graph.

**Build semantic graph.** First, we use a pretrained dependency parser [39] to establish grammatical dependencies between words in the title, as shown in Figure 3. This involves analyzing the sentence structure to identify the relationships between words, such as subject–verb or adjective–noun connections, creating a dependency tree that maps out the grammatical structure of the sentence. Next, we apply a rule-based system [40] to transform the previously obtained dependency tree into a semantic graph. This process begins by identifying key entities such as objects, actions, and attributes within the dependency tree, followed by extracting the relationships between these entities. The rule-based system then converts the grammatical dependencies into a graphical structure, where nodes represent entities like objects, actions, or attributes and edges represent the relationships between them. This results in a semantic graph that visually organizes the sentence’s semantic information, offering a more intuitive and interpretable structure for understanding the complex relationships within the sentence. Such a structured approach is crucial for generating accurate image captions and improving the overall effectiveness of image retrieval systems by matching these graphs against those of available images. This process results in a semantic graph that visually organizes the sentence’s semantic information, providing a more intuitive and interpretable structure for understanding the complex relationships within the sentence. Assuming the sentence is *S*, through the above two steps of processing, we obtain object classes O(s), relation types E(s), and attribute types A(s) from the captions through a semantic analysis:

(13) $$G\left(s\right)=\left\{O\left(s\right),E\left(s\right),A\left(s\right)\right\},$$

O(s) is the object contained in the sentence, $E\left(s\right)\subseteq \left\{O(s)\times R\times O(s)\right\}$ represents the relationship between objects, and A(s) is the property of the object. Similarly, the semantic graph of the sentence only contains the relationships between words and cannot be directly used for dictionary construction. Therefore, it needs to be encoded to adapt to word learning. Since the graphs of images and sentences are similar, the semantic graph of the sentence is compiled in the same way.

**Dictionary.** Unlike the self-attention mechanism used for words in the decoder stage, our dictionary is learned. The role of the dictionary is to re-encode the input word, and when the word is input into the attention mechanism [5], the resulting feature is a fused representation of the input word. However, after re-encoding, it is more likely to produce an encoding of “withered leaf” from “golden and flecked leaf”. Overall, the role of the dictionary is to reconstruct the representation of the input $\hat{x}=R\left(x;D\right)$. Our approach primarily involves utilizing the attention mechanism to build and learn the dictionary **D**.

(14) $$\begin{array}{cc} Q=X_{q}W_{q},K & =X_{k}W_{k},V=X_{v}W_{v},\\ & \Omega_{A}=\mathrm{Softmax}\left(\frac{QK^{⊤}}{\sqrt{D}}\right),\\ & Att=\Omega_{A}V. \end{array}$$

Once the semantic graph encoding is completed, we use the attention mechanism to comprehensively learn the features of the words in the captions to obtain their feature vectors. In this process, $X_{q}$ represents the feature vector of the word while $X_{k}$ and $X_{v}$ represent the encoded semantic graphs. Through the attention mechanism, the newly generated word feature vector integrates the information from the semantic graph.

**Figure 4:**
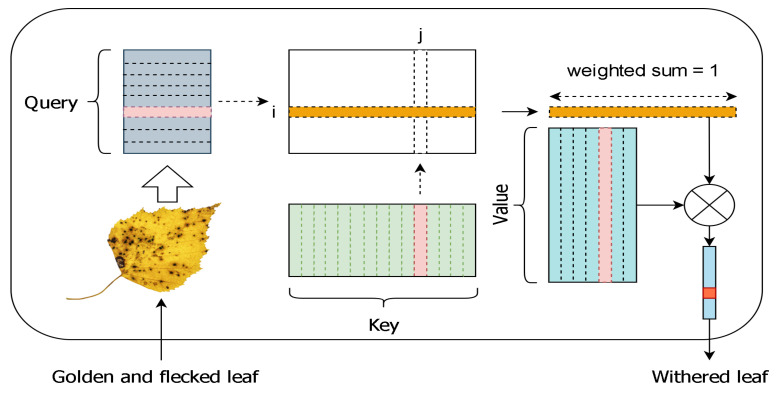
By employing an attention mechanism, the input words are queried in the dictionary to obtain high-level semantic information.

The purpose of **D** is to embed a priori knowledge into language composition. The key role of the dictionary is to reconstruct the feature vectors of the words in the sentence. Similar to DETR’s learnable vectors in the decoding stage, this model creates a fixed number and dimension of learnable features. At this point, we have obtained the word feature vector containing the sentence semantic graph, which serves as the query. The words with n-dimensional trainable feature vectors in the dictionary serve as the keys and values. $W_{q}$, $W_{k}$, and $W_{v}$ are the matrix parameters that need to be learned. The learned words store the high-level features of the captions. As shown in Figure 4, upon completing the dictionary learning, the word features are fed into the dictionary. By querying the keys and aggregating the values with weights, the high-level semantic features of the word are extracted. The calculation process is as shown in Equation (14).

## 4. Experiments

The goal of image captioning is to automatically generate descriptive natural language captions for images by comprehensively understanding their visual content. To achieve this, we set our objective as developing a model capable of generating captions with enhanced accuracy and contextual relevance, addressing the limitations of existing region-based approaches. As introduced in Section 3, our proposed Semantic Scenes Encoder (SSE) model integrates both scene graphs and semantic graphs to capture the spatial relationships and high-level semantic connections within an image, facilitating the generation of more coherent and contextually enriched captions. More details are provided in Section 4.2. For evaluation, we used standard metrics, including BLEU, METEOR, ROUGE, CIDEr, and SPICE, as described in Section 4.3, which collectively assess various aspects of caption quality, such as n-gram precision, semantic accuracy, and syntactic structure. These metrics enable us to evaluate the effectiveness of our approach in generating captions that closely align with human judgment and expectations. To thoroughly validate our model, we conducted a series of experiments on the MSCOCO dataset, detailed in Section 4.1, using both quantitative evaluations and ablation studies to analyze the contributions of different components, such as the scene graph and dictionary module. This multi-step evaluation framework ensures a comprehensive assessment of our model’s performance, robustness, and generalization capabilities across different image scenarios.

### 4.1. Datasets

To systematically assess the effectiveness of our caption model, we carried out a series of experiments utilizing the well-established MSCOCO dataset [42]. MSCOCO stands out as the de facto standard for benchmarking image captioning algorithms, containing a total of 164,062 images. This extensive dataset is subdivided into 82,783 training images, 40,504 validation images, and 40,775 testing images. Each image in the collection is associated with at least five meticulously curated captions, and the annotations for both the training and validation sets are publicly accessible. These extensive annotations make it an invaluable resource for training and evaluation. For the evaluation, we adopted the widely-used Karpathy split [43], which consists of 123,287 images, each accompanied by five annotations. In this split, 113,287 images are allocated for training, 5000 for validation, and another 5000 for testing.

### 4.2. Details

**Preparation work.** Before the experiment begins, the data preprocessing mainly includes four parts: building the model vocab, extracting image features, creating the image scene graph, and constructing the sentence semantic graph.

**Figure 5:**
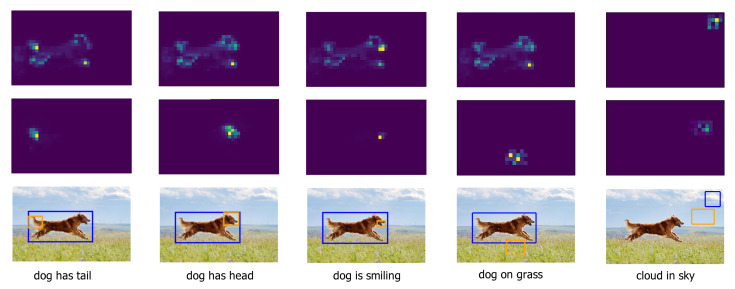
The examples of object relationship visualization in the generation of image scene graph.

- **Vocab** is created using annotations from MSCOCO. Firstly, the words in the sentences are counted to obtain a dictionary of word frequencies. Special characters such as ’.’, ’#’, and ’@’ are then removed, as are words that have a frequency of less than 5. Next, the keywords in the dictionary are converted into a list. Finally, 〈*bos*〉 (begin of sentence), 〈*eos*〉 (end of sentence), 〈*pad*〉 (padding sentence), and 〈*unk*〉 (unknown) are added to the list. The words in the list are then numbered sequentially, resulting in a dictionary where words can be accessed by an index. Through reverse lookup, a dictionary mapping indices to words is obtained. After processing, the total number of words included in the vocabulary is 10,837. During the model’s input phase, the word content is converted into numerical content using the word index dictionary, which is then transformed into vectors for model inference. During the model’s output phase, the numerical content is converted back into words using the dictionary, with indices as keys and words as values.
- **Image feature extraction** is performed using the Faster R-CNN object detection network. We use ResNet-101 as the base model to extract convolutional features from images. Utilizing the RoI Pooling mechanism within Faster R-CNN, a fixed-size proposal feature map (*n* × 2048) is obtained. Subsequently, a fully connected operation is employed for object recognition and localization.
- **Image scene graph** contains information about object relationships and attributes in the image. In this experiment, we use the image region features extracted above as the input for the scene graph generation model. We also trained an attribute classifier using Visual Genome [44]: a small fully connected (fc) softmax network head. As shown in Figure 5, we visualized the features that were focused on during the extraction of the scene graph. The scene graph generated from an image consists of three sets: a set of relationships, a set of objects, and a set of attributes.
- **A semantic graph** not only contains the independent semantics of words but also their explicit and implicit connections. First, captions are parsed into syntactic dependency trees using a Probabilistic Context-Free Grammar (PCFG) dependency parser. Then, a rule-based method is used to map the dependency trees into a semantic graph. After decomposing all the annotations, they are divided into three sets: objects, their relationships, and their attributes.
- **Hardware and software** are also critical components of our experiments. In our implementation, we used a combination of high-performance computing hardware and software libraries to optimize model training and evaluation. Specifically, the model was trained on an NVIDIA RTX 3090ti GPU with 24 GB of memory, allowing for efficient handling of large datasets and the complex computations required for image captioning. The training time for the cross-entropy phase was 6 h and the reinforcement learning phase took 1.5 days. The software environment was built using Python 3.8 and the PyTorch deep learning framework (version 1.9), along with key libraries such as Torchvision for image processing tasks and spaCy for syntactic parsing in semantic graph generation. These components enabled seamless integration of image feature extraction and language modeling processes, ensuring robust, scalable, and efficient model performance.

**Training steps.** The model takes an image as input and first utilizes Faster R-CNN to extract regional features from the image. Subsequently, it uses Motifs to establish relationships between these regions, generating a scene graph. The annotations are then used with SPICE to construct a semantic graph. The scene graph is encoded and fed into the image captioning model’s encoder, while the semantic graph is also encoded and input into a dictionary module to build comprehensive textual information. This information is then passed into the image captioning model’s decoder, ultimately generating the final captions. Therefore, our training process is divided into three steps. Initially, we train a basic image captioning model using both images and the sentence semantic graph. Next, we integrate a dictionary module into the trained model and continue training. Finally, we remove the sentence semantic graph and fine-tune the dictionary to obtain the final model.

**Test steps.** The testing and training phases of the model differ slightly. During both phases, the image serves as the input and the caption as the output. Hence, a trained model can incorporate the scene graph derived from the image but not the semantic graph generated from the caption. Therefore, we trained a dictionary to store the semantic information of the captions. In the training phase, the model takes the image as input, uses Faster R-CNN and Motifs to extract regional features, and constructs a scene graph. This scene graph is then encoded to generate intermediate features. The model uses the intermediate features of the image, along with the information stored in the dictionary, to generate captions.

**Hyperparameters.** We utilize the DLCT [45] as the foundational structure for implementing the entire model. The DLCT introduces a two-tiered attention mechanism that integrates both object-level and global-level features to enhance caption generation. Object-level attention focuses on the fine-grained details of individual objects and their relationships, while global-level attention captures the broader scene context, resulting in more coherent and contextually aware captions. By collaboratively learning from both detailed and high-level representations, the model improves the accuracy and fluency of generated captions, producing richer and more contextually relevant descriptions. All input data vectors are transformed into 512-dimensional embeddings. The optimization process of the experiment is divided into two phases. First, training is performed using cross-entropy loss, as outlined in (2). The Adam optimizer is used to optimize the model parameters, with $\beta_{1}=0.9$, $\beta_{2}=0.98$, and $\varepsilon =10^{-9}$. During this phase, the model undergoes three stages of training, with each stage comprising five epochs at a learning rate of 10^−4^. Afterwards, the learning rate is reduced to 2 × 10^−5^ and another five epochs of training are conducted. In the second phase, the model is fine-tuned using the loss function described in (3), with an initial learning rate of 5 × 10^−6^. Training stops when there is no further improvement in the model performance. For language generation during inference, a beam search strategy is employed with a beam width of five.

### 4.3. Evaluate Method

We conform to the established evaluation protocol for caption generation: BLEU [46], METEOR [47], ROUGE [48], CIDEr [49], and SPICE [22].

BLEU calculates the similarity between n-grams (word sequences) in candidate and reference captions, with distinctions being made for n-grams of lengths ranging from 1 to 4, categorized as BLEU-1 to BLEU-4. METEOR evolved from extending the fundamental method of BLEU to include matches of synonyms, stems, and various morphological changes, aligning more closely with human evaluative standards. Therefore, a high BLEU score indicates precision, while METEOR’s framework, which considers both precision and recall, is better correlated with human judgment of caption quality. ROUGE emphasizes recall and was originally the preferred evaluation metric for automatic caption summarization. Its assessment can describe the extent to which the generated caption contains the key content of the reference caption. ROUGE measures quality by comparing the overlap of words, phrases, and n-grams between the model-generated caption and the reference caption. The higher the score, the better the performance. The evaluation metric specifically designed for image captioning is CIDEr. It primarily calculates the cosine similarity between the TF-IDF vectors of the reference caption and the generated caption, focusing on the consistency among different captions. The weighting process in CIDEr can be expressed as follows:

(15) $$\begin{array}{c} {\mathrm{CIDEr}}_{n}\left(c_{i},r_{i}\right)=\frac{1}{m}\sum_{j}\frac{g^{n}\left(c_{i}\right)g^{n}\left(r_{ij}\right)}{∥g^{n}\left(c_{i}\right)∥∥g^{n}\left(r_{ij}\right)∥},\\ \mathrm{CIDEr}\left(c_{i},r_{i}\right)=\sum_{n=1}^{N}\omega_{n}{\mathrm{CIDEr}}_{n}\left(c_{i},r_{i}\right), \end{array}$$

where $r_{i}$ denotes the reference annotation and $c_{i}$ represents the candidate sentence. The vector $g^{n}$ includes all n-grams of length n. SPICE prioritizes the semantic content of captions over simple lexical matches, leading to a more precise assessment of their semantic quality. To thoroughly evaluate the reliability of the task, it is common to combine SPICE with other evaluation metrics.

## 5. Results and Ablation Study

We first discuss the experimental results. Then we conduct several ablation experiments to evaluate the impact of various components and parameters in this approach. First, since a sentence semantic graph involves encoding words, we analyzed the number of words in the annotations and assessed how different vocabulary sizes affect model performance. Next, to validate the contributions of the two components—the image scene graph and the sentence semantic graph—we carried out ablation experiments. The amount of information stored varies with dictionary size; therefore, we conducted further ablation tests to evaluate the performance of different numbers of feature vectors and analyzed the results accordingly.

### 5.1. Experiments Results

Table 1 shows the performance of the SSE method on the test set under the Karpathy [43] split. First, we evaluated the models using the BLEU [46] metric, and the results showed that the SSE outperformed others in BLEU-1 and BLEU-4. This success is attributed to our dictionary, which integrates textual information from captions, allowing the model to leverage the rich semantic information extracted from annotations. Metrics such as ROUGE [48] and METEOR [47], similar to BLEU, focus on individual word matches. The integration of our dictionary significantly enhances the model’s performance in these metrics. CIDEr [49], which combines elements of BLEU with vector space models, benefits from a comprehensive evaluation method. The dictionary allows the model to effectively leverage the rich semantic information, improving performance across these varied metrics. SPICE [22] considers extensive information about the objects, attributes, and relationships within the image. By incorporating a scene graph of the image, we can better describe the positional relationships of the objects, leading to a superior performance in SPICE. Consequently, the SSE achieves the best scores in both CIDEr and SPICE. Compared to the baseline model DLCT, the model enhanced with the SSE method demonstrated improvement across all five evaluation metrics. Notably, in the CIDEr metric, the SSE showed a 2.6% increase, improving from 133.8 in the baseline DLCT [45] to 137.4.

The advantage of the SSE is not only reflected in improved evaluation metrics; the true advantage of our approach lies in its ability to integrate both scene graphs and semantic graphs. This integration allows for a more comprehensive understanding of both spatial relationships and high-level semantic connections during the image captioning process. Unlike existing models, which often focus solely on visual features or rely heavily on predefined memory components, our model dynamically incorporates contextual information from both the visual content and the semantic structure of captions. This results in more coherent and contextually relevant captions, especially for complex scenes where relationships between objects are critical. Additionally, by leveraging a learnable dictionary to store high-level semantic information, our model can better capture nuances in image descriptions, thus improving both accuracy and diversity in the generated captions.

### 5.2. Vocab

**Figure 6:**
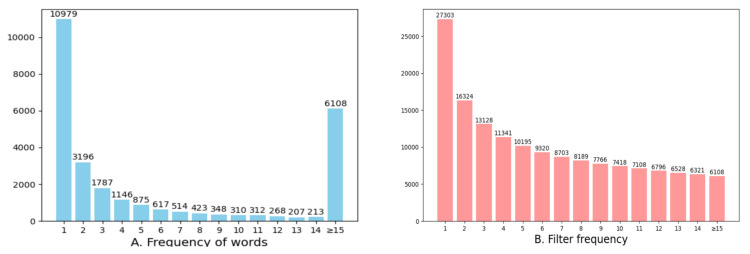
The image summarizes the word statistics in the vocabulary.

Although a larger vocabulary allows for more descriptive words in image captions, it also increases the difficulty of model convergence. During caption encoding, words are represented as one-hot vectors, and these are then transformed into the model’s internal vector dimensions via embeddings. When decoding the caption, softmax is applied to select the word with the highest probability. However, with a larger vocabulary, the probability distribution becomes more dispersed, making the model harder to train. Consequently, this section examines the impact of vocabulary size on model performance. To investigate this, we first split all captions into individual words using Spacy’s tokenizer. Each word is processed and counted to create a comprehensive dictionary with their frequencies. The resulting dictionary contains a total of 27,303 words, as illustrated in Figure 6. Given that the number of words in the dictionary can affect the model’s performance, we conducted a statistical analysis of the word distribution and evaluated models containing words of varying frequencies.

**Figure 7:**
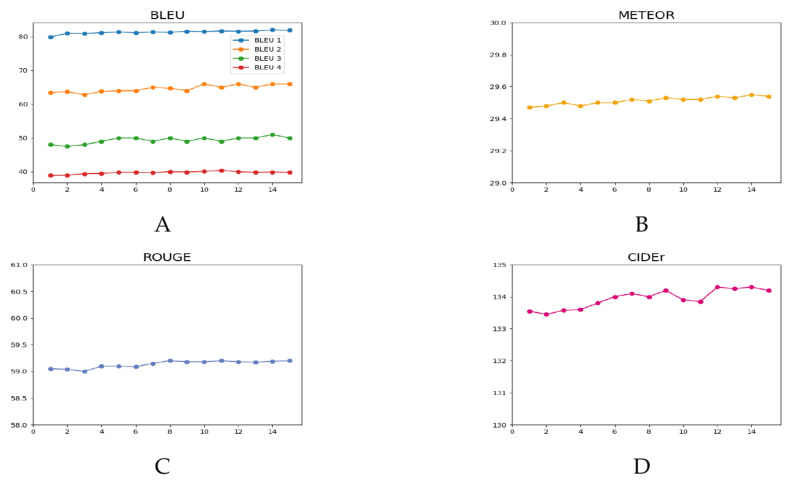
The model’s performance at different frequencies. (**A**) represents BLEU. (**B**) represents METEOR. (**C**) represents ROUGE. (**D**) represents CIDEr.

We summarized the frequency distribution of the words in the annotations and consolidated the number of word occurrences to represent the total number of words in the vocabulary at different filtering frequencies. For example, the fifth bar indicates that 875 words appeared exactly five times, and the total number of words with a frequency of at least five is 10,195. Words with a frequency exceeding 14 times were combined, totaling 6108. In this ablation experiment, we set different retention frequencies, resulting in vocabulary sizes of 27,303, 16,324, 13,128, etc. The results, shown in Figure 7, demonstrate that, as the filtering frequency increases, the reduction in the number of words leads to a gradual improvement in experimental scores. These findings support our hypothesis that having fewer words makes it easier for the model to converge.

### 5.3. Components

The SSE includes two modules: the image scene graph and the dictionary. To evaluate the performance of the image scene graph and the dictionary unit, we tested them separately. The results are shown in Table 2.

Table 2 shows that both modules improve the model’s performance. When the model uses both modules simultaneously, the performance improvement exceeds a simple linear combination. We believe this is because the inclusion of an image scene graph enriches the information stored in the dictionary, resulting in generated captions that contain the structural information of the images.

#### Different Number of Dictionary

The ablation study removes the image scene graph, retaining only the dictionary. We first trained the model with cross-entropy loss before subsequently training it using self-critical loss to obtain the final results. The experimental results are shown in Table 3. When the number of entries stored in the dictionary is 150 or 200, the model achieves an optimal performance, contrary to the expectation that more entries would yield better results. This phenomenon can be attributed to the following reasons:

1. Information Density: A sparse dictionary with too few entries fails to encapsulate adequate information, resulting in an incomplete representation of visual features.
2. Attention Distribution: An excessive number of dictionary entries leads to attention diffusion, where significant features are diluted by redundant ones.
3. Training Stability: An optimal number of entries strikes a balance by providing sufficient information without overwhelming the model, thereby maintaining stability during training and the caption generation process.

### 5.4. Limitations and Practical Applications

While the Semantic Scenes Encoder (SSE) demonstrates significant improvements in regard to image captioning, there are notable limitations that must be addressed. First, the SSE relies heavily on accurate scene graph generation, which is not always feasible for complex or highly abstract images. Misrepresentation in the scene graph could lead to erroneous captions, particularly in scenarios where objects and relationships are not well defined. Additionally, the construction of semantic graphs from captions introduces complexity, which may not be practical in low-resource environments due to the increased computational demand. This limitation could hinder real-time applications and restrict the scalability of the model to larger datasets or more diverse image domains.

The model’s performance also depends on high-quality annotated data, such as MSCOCO, and its effectiveness might diminish when faced with images outside the scope of such datasets. In addition, while reinforcement learning enhances caption quality, it introduces challenges related to fine-tuning the reward structure, which can lead to convergence issues if not properly handled. The lack of exploration into lightweight versions of the SSE also suggests that the current model might be too resource-intensive for certain practical applications. Despite these limitations, the SSE model holds significant promise for various real-world applications. In healthcare, for example, it could be used to generate descriptive reports from medical images, like CT [54,55] or MRI scans [56], provided that the model is trained with domain-specific data. Its ability to extract relationships between objects and generate contextually rich captions could assist radiologists in interpreting medical imagery by highlighting critical findings in a structured format. This would potentially streamline the diagnosis process and reduce human error. In fields like autonomous driving, the SSE model could enhance scene understanding by generating detailed descriptions of complex traffic environments. This capability would be invaluable for improving decision-making systems in self-driving cars, enabling them to respond more effectively to dynamic road conditions. Similarly, in e-commerce, the SSE could improve product recommendations by generating precise product descriptions based on user-uploaded images.

Societal impact can be further amplified through the creation and use of well-annotated datasets specific to diverse applications. However, the model has not yet been tested on datasets outside standard benchmarks like MSCOCO. We plan to explore whether a domain-specific dataset for healthcare or autonomous driving could further validate the model’s robustness. Without such evaluations, the model’s broader scientific contributions could remain limited. Moving forward, expanding the scope of this work to include new datasets will be crucial for positioning the SSE as a versatile tool across industries. In conclusion, the SSE offers substantial improvements in image captioning, but further refinement and evaluation in diverse contexts are necessary to fully realize its potential in real-world applications.

## 6. Conclusions and Future Work

In conclusion, this paper introduces the Semantic Scenes Encoder (SSE), a novel framework that integrates image scene graphs and semantic graphs into the process of image captioning. By converting images into a scene graph and utilizing SPICE to transform captions into a semantic graph, the SSE effectively leverages these semantic representations to train a word dictionary. The effectiveness of these integrated components has been validated, with ablation experiments confirming that both significantly enhance the model’s performance. However, the approach’s success is contingent on the accuracy of the scene graph generation, and its complexity may result in increased computational demands.

Looking ahead, future research could explore more complex scene and semantic graph construction methods to capture richer information from images and captions, thereby enhancing the model’s understanding capabilities. Additionally, integrating the SSE with other advanced natural language processing techniques, such as large language models or self-supervised learning, could further improve the accuracy and fluency of generated captions. Finally, the application of the SSE is not limited to image captioning; it can also be extended to other tasks, such as visual Qqestion answering (VQA), image retrieval, and video understanding.

## Figures and Tables

**Table 1:** The performance of SSE compared to the others is superior.

Methods	BLEU-1	BLEU-4	METEOR	ROUGE	CIDEr	SPICE
SCST [38]	-	34.2	26.7	55.7	114.0	-
BUTD [37]	79.8	36.3	27.7	56.9	120.1	21.4
ORT [50]	80.5	38.6	28.7	58.4	128.3	22.6
AoANet [51]	80.2	38.9	29.2	58.8	129.8	22.4
*M*^2^ [9]	80.8	39.1	29.2	58.6	131.2	22.6
CMTVC [52]	81.5	39.7	**30.0**	59.5	135.9	23.7
DSNT [53]	81.5	39.9	29.5	59.2	134.8	23.1
DLCT [45]	81.4	39.8	29.5	59.1	133.8	23.0
SSE	81.6	40.8	30.0	59.7	137.4	23.8

**Table 2:** Model performance using different components.

Trans	Isg	Memory	BLEU1	BLEU4	METEOR	ROUGE	CIDEr
✓			81.4	39.8	29.5	59.1	133.8
✓	✓		81.2	39.9	29.7	59.4	134.7
✓		✓	81.5	40.9	29.6	59.7	135.9
✓	✓	✓	81.6	40.8	30.0	59.7	137.4

**Table 3:** Performance with different numbers of feature vectors in the dictionary.

N	Cross-Entropy Loss				Self-Critical Loss			
	BLUE1	METEOR	ROUGE	CIDEr	BLUE1	METEOR	ROUGE	CIDEr
50	75.2	28.7	57.7	117.0	80.9	29.4	59.4	134.1
100	75.0	28.5	57.8	117.2	81.0	29.5	59.6	135.2
150	75.4	29.0	58.1	118.5	81.6	30.0	59.7	137.4
200	75.9	29.1	58.1	118.2	81.7	29.9	59.6	137.0
250	76.3	28.8	58.3	118.0	81.4	29.8	59.5	136.3
300	76.2	28.9	58.0	117.9	81.6	29.6	59.5	135.4

## Data Availability

The MSCOCO dataset is publicly available and can be downloaded from the https://cocodataset.org, accessed on 1 April 2015. The dataset used in this paper can be accessed at https://pan.baidu.com/s/1scmeYCVAbjFCG1B8li6Z2Q?pwd=8e8s. The base model code used in this paper is available at https://github.com/luo3300612/image-captioning-DLCT. The addresses for the proposed image scene graph and semantic graph processing modules in this paper are available at https://github.com/PhoenixZhi/sentence-graph.
